# Fluorescence Enhancement of Fluorescein Isothiocyanate-Labeled Protein A Caused by Affinity Binding with Immunoglobulin G in Bovine Plasma

**DOI:** 10.3390/s91008271

**Published:** 2009-10-20

**Authors:** Takehito Ogawa, Satoka Aoyagi, Takehiro Miyasaka, Kiyotaka Sakai

**Affiliations:** 1 Department of Chemical Engineering, Waseda University / 3-4-1 Okubo, Shinjuku-ku, Tokyo 169-8555, Japan; E-Mail: t-ogawa@aoni.waseda.jp (T.O.); 2 Faculty of Life and Environmental Science, Shimane University / 1060 Nishikawatsu-cho, Matsue-shi, Shimane 690-8504, Japan; E-Mail: aoyagi@life.shimane-u.ac.jp (S.A.); 3 Department of Medical Engineering, Himeji Dokkyo University / 7-2-1 Kamiono, Himeji-shi, Hyogo 670-8524, Japan; E-Mail: miyasaka@himeji-du.ac.jp (T.M.)

**Keywords:** fluorescence enhancement, immunoassay, immunoglobulin G, Protein A

## Abstract

Fluorescence enhancement of fluorescein isothiocyanate-labeled protein A (FITC-protein A) caused by the binding with immunoglobulin G (IgG) in bovine plasma was studied. FITC-protein A was immobilized onto a glass surface by covalent bonds. An increase in fluorescence intensity was dependent on IgG concentration ranging from 20 to 78 μg/mL in both phosphate buffer saline and bovine plasma. This method requires no separation procedure, and the reaction time is less than 15 min. A fluorescence enhancement assay by the affinity binding of fluorescence-labeled reagent is thus available for the rapid determination of biomolecules in plasma.

## 1. Introduction

Fluorescence measurement has been widely used in immunoassay and in determination of bio-interactions. Determination of fluorescence properties enables analysis of micro-environmental changes near fluorescence dyes and biomolecular interactions [1-3]. Progress in optical devices provides a convenient and portable measuring system using a diode laser as light source and a charge-coupled device (CCD) as fluorescence detector [4]. These improvements make it possible to apply fluorescence assays to on-site and rapid measuring systems.

Some authors have reported the measurement of biomolecules and biologically active compounds by fluorescent enhancement [5-7]. This method is based on the enhanced fluorescence intensity of a fluorescent-labeled reagent which interacts with a target substance. The affinity binding of the target substance with the labeled reagent provides a hydrophobic microenvironment near the fluorescence label, which results in the enhancement of fluorescence. The determination of biomolecules by fluorescence enhancement has advantages of convenience and speed.

In the application of fluorescence enhancement in immunoassay, the time of determination is shortened to less than 5 min because of the separation-free and reagent-less method [8]. Also this simple method is independent of operator skill, so its application to *in situ* measurements, like preliminary diagnostics or environmental measurements, is expected. For these purposes, less pretreatment of sample solutions is required.

The objective of the present study is to evaluate fluorescent enhancement of a fluorescein isothiocyanate-labeled protein A by the affinity binding with immunoglobulin G in bovine plasma. Application of this fluorescence enhancement immunoassay for plasma samples is discussed in comparison with the fluorescence in buffer solution.

## 2. Experimental Section

### 2.1. Materials

Chemicals were purchased from Wako Pure Chemicals Industry (Osaka, Japan) unless otherwise mentioned. Toluene was used after dehydration with molecular sieves (4 Å). Bovine plasma was obtained by centrifugation from bovine blood containing anticoagulant (hematocrit: 30.0%–38.4%, total protein: 8.13–9.08 g/dL, anticoagulant: 10 vol% of 3.2 w/v% sodium citrate). Stock solution of immunoglobulin G (IgG) was prepared by diluting rabbit IgG purchased from Nordic Immunological Laboratories (Tilburg, Netherlands). Sample IgG from rabbit is known to bind with protein A strongly. Bovine plasma was selected as bulk solvent for the ease of use and weak binding ability of bovine IgG to protein A. Fluorescein isothiocyanate (FITC) was used for the fluorescence label because the fluorescence enhancement of FITC caused by micro-environmental change has been well established by many authors [7-9]. Other regents were used without further purification.

### 2.2. Immobilization of FITC-labeled protein A

**Scheme 1.**
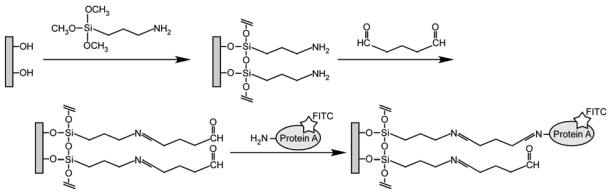
Procedure for immobilization of FITC-labeled protein A.

A glass slide (8 mm × 8 mm) was reacted with 3.8 vol% 3-aminopropyltrimethoxysilane (Tokyo Chemical Industry, Tokyo, Japan) in dehydrated toluene at 383 K for 6 hr. The silanized glass was immersed in 1% glutaraldehyde solution for 1 hr. FITC-labeled protein A (FITC-protein A; Zymed Laboratories, South San Francisco, CA, USA) was immobilized onto the glass by immersing the glass in a phosphate buffer saline (PBS, pH 7.4) containing 25 μg/mL FITC-protein A for 40 hr. The procedure for immobilization of FITC-protein A is shown in Scheme 1. After FITC-protein A was immobilized, the glass slides were treated in dark. The FITC-protein A immobilized glass was immersed in 0.5 g/L bovine serum albumin (BSA) solution for 1 hr to block nonspecific adsorption. The glass was stored in PBS at 277 K.

### 2.3. Fluorescence intensity measurement

**Figure 1.**
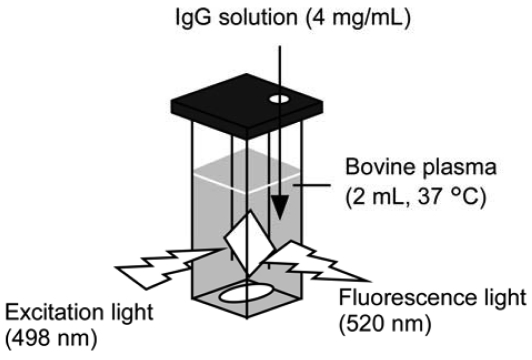
Experimental setup for measurement of fluorescence intensity.

The glass slide was located in a plastic cell containing 2 mL buffer or 2 mL bovine plasma solution as shown in Figure 1. IgG concentration of sample solution was adjusted by adding 10–40 μL of 4 mg/mL IgG stock solution to sample solvent in the cell. Fluorescence intensity was measured at an excitation wavelength of 498 nm on spectrofluorophotometer (RF-5300PC, Shimadzu, Kyoto, Japan). The excitation light was irradiated at an incident angle of over 45° to glass surface, and emission light was collected near a perpendicular line to separate emission light from reflection light. The average value for fluorescence intensities from 519 nm to 521 nm at steady state was used for analysis of IgG concentration dependency, whereas the integration of the intensity was used in previous report [8]. Experiments of fluorescence intensity were performed at 310 K under stirring.

## 3. Results and Discussion

### 3.1. Fluorescence spectrum of FITC-protein A immobilized glass

**Figure 2.**
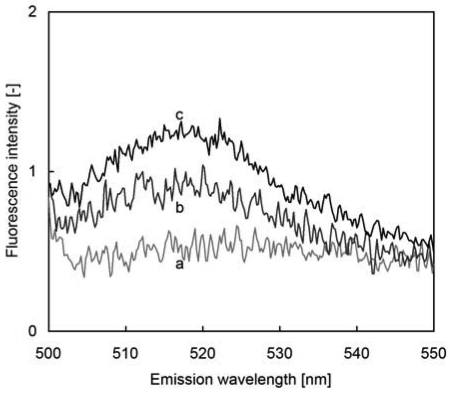
Fluorescence spectrum of immobilized FITC-Protein A. Line ‘a’ is spectrum of bovine plasma without FITC-Protein A immobilized glass, Line ‘b’ is spectrum of immobilized FITC-Protein A immersed in bovine plasma, Line ‘c’ is enhanced fluorescence spectrum at 78 μg/mL IgG in bovine plasma.

Fluorescence spectra of FITC-protein A immobilized glass in bovine plasma are shown in Figure 2. Without the glass, broad band emission of bovine plasma was observed (line a in Figure 2). This fluorescence is emitted in the presence of plasma protein and bilirubin [10]. After setting the FITC-protein A immobilized glass in the plastic cell, a maximum fluorescence intensity was observed at 520 nm (line b in Figure 2). The maximum fluorescence intensity with FITC in PBS at 520 nm indicates that the FITC-protein A is immobilized on the glass [8]. By addition of IgG up to 78 μg/mL, FITC emission at 520 nm was enhanced (line c in Figure 2). Since a steady state of fluorescence intensity was reached at 15 min after addition of IgG solution, IgG measurements were performed after 15 min.

To determine whether the fluorescence enhancement was caused by the native plasma protein or immobilized FITC-protein A, fluorescence spectrum of plasma solution without FITC-protein A immobilized glass was measured. Fluorescence spectrum was unchanged by adding IgG in the absence of FITC-protein A glass. Fluorescence enhancement shown in Figure 2 results from immobilized FITC-protein A.

### 3.2. Dependency of fluorescence enhancement on IgG concentration

**Figure 3.**
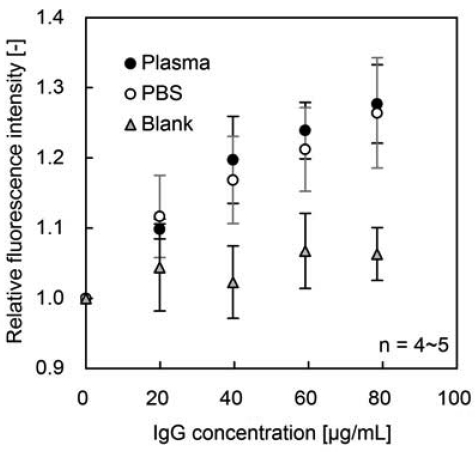
Dependency of relative fluorescence intensity on IgG concentration. Black circles denote FITC-protein A immobilized glass in bovine plasma, White circles denote FITC-protein A immobilized glass in PBS, Gray triangles denote plasma solution without glass for blank test.

Differences in the amount of immobilized FITC-protein A and in the FITC/protein A molar ratio cause various fluorescence intensities of immobilized FITC-protein A for the test glasses. To correct the individual variability, relative fluorescence intensity, ratio of fluorescence intensity after IgG addition to that before IgG addition, was used. Dependence of relative fluorescence intensity on IgG concentration is shown in Figure 3. Relative fluorescence intensity increased with IgG concentration, and reached approximately 1.3 at 78 μg/mL IgG in bovine plasma. Relative fluorescence intensity gave a good correlation between PBS and bovine plasma. IgG in bovine plasma is hence measurable by fluorescence enhancement of immobilized FITC-protein A.

It has been reported that the degree of fluorescence enhancement of FITC caused by affinity binding was 1.6-fold with protein A — IgG interaction in PBS [8] and 1.5-fold with DNA interaction [7]. On the other hand, glycerin treatment enhances fluorescence of immobilized FITC-labeled antibody by approximately 1.5-fold [9]. The lower fluorescence enhancement shown in Figure 3, which may be caused by differences in purification or preservation of the purchased reagents, leaves much room for fluorescence enhancement at higher IgG concentrations.

There are some reasons accounting for fluorescence enhancement of fluorescent label, such as the microenvironment of fluorescence dye, viscosity and polarity of solvent [11-13]. Fluorescence enhancement caused by affinity binding may result from the hydrophobic microenvironment [7,8]. The fluorescence enhancement of FITC-protein A after binding with IgG in bovine plasma gave almost the same relative fluorescence intensity as that in PBS, demonstrating relatively low difference in solvent effect on fluorescence enhancement between buffer solution and plasma.

According to the Student's *t*-test, there was a significant difference in relative fluorescence intensity between 0 and 20 μg/mL IgG in bovine plasma (P < 0.05, Figure 3). Hence IgG is detectable by the fluorescence enhancement at over 20 μg/mL IgG in bovine plasma. However no significant difference was observed between plasma measurement and blank test at 20 μg/mL IgG (P > 0.05). It seems to be caused by the dispersion of FITC-Protein A-immobilization in each sample glass and plasma-related noise for fluorescence. More sensitive measurement will be achieved by the further improvement in immobilization method and choice of fluorescence dye, because fluorescence wavelength of FITC is overlapped with that of plasma.

Application of the fluorescence enhancement method in the determination of edaravone [6] and dioxins [7] has been reported. This method has a potential applicability to measure substrates which interact with other molecules. Most expected applications are to determine antigens using labeled-antibodies and also antibodies using labeled-antigens. Fluorescence enhancement after an antigen-antibody reaction depends on the molecular weight and hydrophobicity of the antigen and also a method of labeling antigen and antibody.

Because measurement of fluorescence enhancement using the fluorescence reagent-immobilized glass is a reagent-less and non-separation method, it has advantages for real time and on-site measurements. This method is applicable to the case of bovine plasma, and the measurement time is approximately 15 min. Such a simple and convenient assay is useful for *in situ* and diagnostic measurements.

## 4. Conclusions

Fluorescence of FITC-protein A in bovine plasma increases with IgG concentration. Extent of fluorescence enhancement in bovine plasma is almost the same as that in PBS. We are able to determine IgG in bovine plasma at 20–78 μg/mL within a reaction time of 15 min. The fluorescence enhancement assay is available for convenient and rapid determination of biomolecules in plasma.
